# Multi-Scale Study of the Small-Strain Damping Ratio of Fiber-Sand Composites

**DOI:** 10.3390/polym13152476

**Published:** 2021-07-27

**Authors:** Haiwen Li, Sathwik S. Kasyap, Kostas Senetakis

**Affiliations:** 1 Faculty of Civil Engineering and Geosciences, Delft University of Technology, 2628 CN Delft, The Netherlands; h.li-11@tudelft.nl; 2 Department of Architecture and Civil Engineering, City University of Hong Kong, Kowloon Tong, Hong Kong, China; ssarvadev2-c@my.cityu.edu.hk

**Keywords:** ground treatment, geosynthetics, fibers, damping, energy dissipation, resonant column testing

## Abstract

The use of polypropylene fibers as a geosynthetic in infrastructures is a promising ground treatment method with applications in the enhancement of the bearing capacity of foundations, slope rehabilitation, strengthening of backfills, as well as the improvement of the seismic behavior of geo-systems. Despite the large number of studies published in the literature investigating the properties of fiber-reinforced soils, less attention has been given in the evaluation of the dynamic properties of these composites, especially in examining damping characteristics and the influence of fiber inclusion and content. In the present study, the effect of polypropylene fiber inclusion on the small-strain damping ratio of sands with different gradations and various particle shapes was investigated through resonant column (macroscopic) experiments. The macroscopic test results suggested that the damping ratio of the mixtures tended to increase with increasing fiber content. Accordingly, a new expression was proposed which considers the influence of fiber content in the estimation of the small-strain damping of polypropylene fiber-sand mixtures and it can be complementary of damping modeling from small-to-medium strains based on previously developed expressions in the regime of medium strains. Additional insights were attempted to be obtained on the energy dissipation and contribution of fibers of these composite materials by performing grain-scale tests which further supported the macroscopic experimental test results. It was also attempted to interpret, based on the grain-scale tests results, the influence of fiber inclusion in a wide spectrum of properties for fiber-reinforced sands providing some general inferences on the contribution of polypropylene fibers on the constitutive behavior of granular materials.

## 1. Introduction

Ground improvement using fibers as a geosynthetic has received significant interest in geotechnical engineering research and practice. Applications of synthetic (or natural) fibers as a means of soil reinforcement may refer in the rehabilitation of locally failed slopes, the increase of the bearing capacity of weak soils, their use in backfill and embankment strengthening and the enhancement of their energy dissipation capacity, or in the mitigation of piping problems in hydraulic structures and preventing erosion [1,2,3,4,5,6,7,8,9,10,11,12,13].

Based on the current state-of-the-art, the use of fibers as a means of ground improvement is in general favorable for large deformation problems, i.e., increasing the shear strength of soils at peak and critical states, or improving the liquefaction resistance of soils [5,14,15,16]. However, it is more conflicting the influence of fibers on the performance of soils at smaller strains. For example, the recent studies by Li and Senetakis [17] and Li et al. [18] showed that at very small strains, the addition of polypropylene fibers reduces the stiffness of sands, thus the favorable or unfavorable application of fibers will depend on the specific application and whether improvement of the small or large-deformation behavior of soils is the major target. Despite this, a relatively limited number of studies have investigated the properties of fiber-sand mixtures in the regimes of small and small-to-medium strains over the previous years [1,15,17,18,19,20,21,22,23], as most works focused on their large-deformation behavior. Recent published data by Li and Senetakis [23] and Li et al. [1] showed that despite the decrease of small-strain stiffness of soils when fibers are added, the stiffness reduction and damping increase curves become more linear in shape, which is contributed, predominantly, by the increased linearity of the stress-strain curves of fiber-reinforced soils as the tensile resistance of fibers is mobilized against the induced strains predominantly beyond the elastic-linear regime. Thus, proper modeling of fiber-soil mixtures necessitates more systematic studies to be performed targeting given ranges of strains (i.e., small-strain, small-to-medium strain, and large-deformation regimes) and given properties. In this direction, the present study attempted to contribute majorly in the analysis of sand-fiber mixtures at small-strains with emphasis on material damping characteristics.

In the analysis of geotechnical engineering systems subjected to vibrations (for example because of seismic-induced loads), modeling of soils (and geosynthetics) is typically performed by analyzing stiffness and damping ratio in the regimes of small and small-to-medium strains [24,25,26,27,28,29], typically between about 10^−4^% to 10^−1^%. Particularly for the measurement (and modeling) of damping ratio, one of the most common laboratory methods is the resonant column which allows the assessment of this property in a wide range of strains (from small-to-medium strain amplitudes). Resonant column testing is also an adequate method in characterizing material behavior in terms of shear modulus and modulus reduction curves. Previous studies investigated the damping ratio of sands and have concluded that major influencing factors are the type of the material which affects the grain-contact response (i.e., particle-to-particle interactions), as well as the grain size distribution curve, the particle shape and the confining pressure/stress ratio as some of the most important parameters which control soil dynamic behavior [29,30,31,32,33,34,35,36]. It is important to notice that in soil dynamics research and practice, such as in the simulation of ground vibrations or soil-structure interaction problems, material properties are commonly modeled based on the normalized modulus against strain and damping ratio against strain curves (G/G_max_ - γ and D_s_ - γ), where G and G_max_ are the “nonlinear” shear modulus and maximum shear modulus (corresponding to small strains), respectively, D_s_ is the damping ratio in shear and γ is the shear strain amplitude. Specifically, hyperbolic types of models are used to model the normalized shear modulus, while damping is commonly modeled based on inter-correlation with shear modulus reduction curves. Additionally, it is a common practice in the development of semi-analytical or empirical expressions, to simulate damping ratio curves based on normalized values, in the form of (D_s_ − D_s,min_) against strain or (D_s_/D_s,min_) against strain [28,29,32,36], where D_s,min_ is the damping ratio at small strains. This means that in order to properly model the medium-strain dynamic properties, small-strain parameters (G_max_, D_s,min_) must be defined, which are commonly expressed through “power-law” types of expressions as a function of confining pressure and other influencing factors [28,29,30,31,33,34,35,36,37]. These properties and developed expressions (both at small and medium strains) are directly used in computer codes for seismic response analysis, for example software packages which use the equivalent linear approach and iteration steps in 1-dimensional ground response studies, or codes which use the finite element method [38,39,40,41]. Clayton [42] demonstrated that nonlinear soil properties (specifically normalized shear modulus and the pressure-dependency of shear modulus) are essential to be modeled also in the analysis of static problems, as for a majority of geotechnical engineering infrastructures it is critical that they are analyzed at strains well below the regime of peak strength (or at stresses and strains which cause failure), while soil dynamic properties are also essential to be modeled (apart from design purposes) in the characterization of sediments with applications from engineering seismology and applied geophysics to petroleum exploration [25,26,27,43]. Within this context, measurements of small-strain damping ratio of any geomaterial (or geosynthetic system) are important to be obtained for complete modeling; however, despite the vast majority of studies in the literature focusing on small-to-medium strain shear modulus, studies on the damping of geomaterials and geosynthetics are rather limited, which was one of the motivations behind this work.

Despite the significant insights obtained in some of the previous studies which examined (and modeled) the small-strain damping of soils, there are many unresearched areas, for example the understanding of the involved micromechanical (or grain-scale) mechanisms which contribute to the energy dissipation of granular materials, and the relative literature with respect to composite systems such as fiber-reinforced sands is very limited. Preliminary studies showed that in general, the damping ratio of sands increases when fibers are added [17]; however this problem has not been studied by examining a broad range of sands with different particle shapes and grading characteristics, which are important influencing factors affecting the behavior of pure sands and, as the literature would suggest, the dynamic properties of fiber-sand composites as well [18]. Additionally, even though there is a general consensus in the literature that the bulk behavior of geomaterials and binary mixtures is firmly linked to grain-scale parameters, there is very limited number of works in the current state-of-the-art to provide experimental data which may contribute to a better linkage between macro- and micro-scale responses of granular systems, which is particularly true for soils reinforced with fibrous geosynthetics. Previous studies, have implemented micro-CT Xray tomography analysis in the study of fiber-reinforced soils [44] providing important insights into the micromechanisms of composite materials; however the area of small (and small-to-medium) strains is highly unexplored in terms of multi-scale insights, which is one of the major new contributions from the present work.

The present study attempted to investigate and model, based on semi-analytical correlations, the influence of fiber content on the small-strain damping ratio of sands taking into account a large number of parameters and by performing resonant column tests on isotropically consolidated samples. Additional insights were attempted to be obtained by performing grain-scale tests on particle-to-particle contacts with and without fibers to interfere in the contact region of the sand grains. These particle-to-particle experiments were performed in an attempt to provide some additional insights on the contribution of fibers in the contact response of sand grains, which might have an influence on the bulk behavior of the composite material. Despite this, the grain-scale tests attempted, majorly, to provide some qualitative inferences on the potential role of fiber inclusion on the damping ratio as obtained from the macroscopic tests, as there was not a direct link of fiber percentage from the resonant column test samples with that of the grain-scale tests. Thus, the main new contributions from the present study to the state-of-the-art are summarized as follows (i) Thorough analysis of the small-strain damping of polypropylene fiber-sand composite materials, taking into account the sand particle size (and shape) characteristics and (ii) Providing a multi-scale analysis of the (potential) energy dissipation mechanisms of fiber-reinforced sand. Results from the present study could comprise a firm basis also in future research, for example taking proper calibrations and input parameters (both microscopic and macroscopic parameters) in discrete-based numerical simulations, which in conjunction with micro-CT Xray tomography can provide direct multi-scale insights on the behavior of materials as a bulk, which was not feasible with the experimental setups of the present work (i.e., grain-scale and resonant column tests were complementary but performed on independent samples).

## 2. Materials and Methods

### 2.1. Test Materials

The macroscopic experiments involved 12 different sands and polypropylene fibers were used as the geosynthetic reinforcing material to create the composite fiber-sand mixtures. The host sands consisted of three types of soils (Sydney sand, Blue sand and White sand) with different origins: (i) Sydney sand (denoted as SS) is a poorly graded natural quartz sand with regular-in-shape particles; (ii) Blue sand (denoted as BS) consists of a basaltic crushed rock of irregular-in-shape particles; (iii) White sand (denoted as WS) is a poorly graded natural quartz sand of regular-in-shape grains (WS has slightly more regular-in-shape grains compared with the SS sand). The specific gravity of solids for all these materials was found to be equal to 2.65 (note that all the sands are silica-based geological materials). Previous studies provided descriptions and characterizations of these different sands in terms of particle shape characteristics and macroscopic behavior [17,35,36,37]. These materials and their respective reconstituted fractions represent a broad range of sands used as earth materials in infrastructure projects.

**Figure 1:**
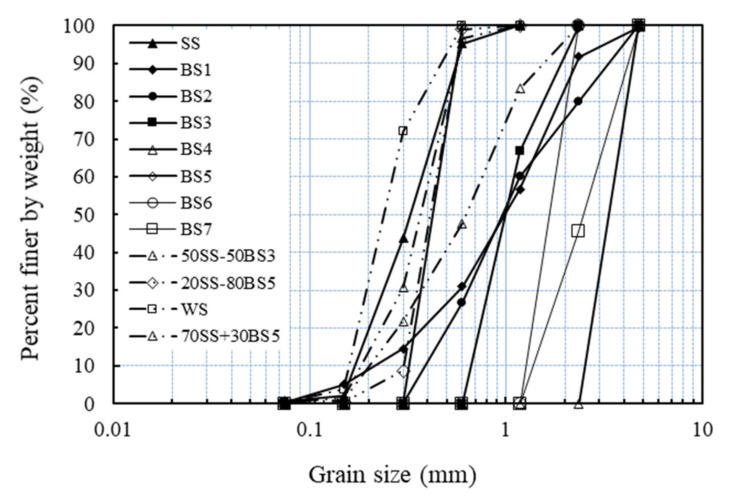
Particle size distribution curves of tested sands.

From these three different host sands, artificial samples were created in the laboratory so that to cover a wide range of grain size distribution characteristics and particle shapes. Specifically, SS and WS were tested (and mixed with fibers) in their natural state, while from the Blue sand, seven different gradings were constructed (denoted as BS1 to BS7). Additionally, different fractions from the Blue sand were mixed with the Sydney sand and the White sand at different proportions to form artificial samples with mixed particle shapes. The grading curves of the 12 sands are illustrated in Figure 1, while Table 1 gives a summary of the different laboratory created samples and their grain size and particle shape characteristics.

**Figure 2:**
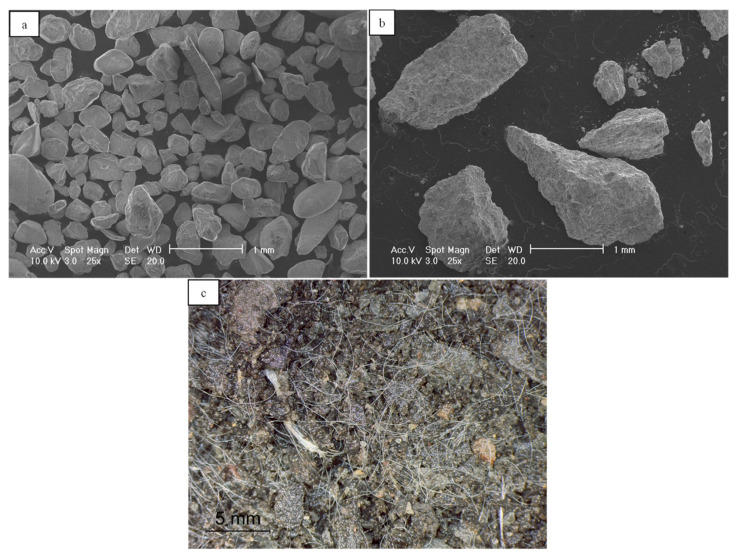
Scanning electron microscope (SEM) images of (**a**) Sydney sand, (**b**) Blue sand 1, and (**c**) Blue sand 1 mixed with polypropylene fibers.

Scanning electron microscope (SEM) images of representative samples from these materials are given in Figure 2a,b. Note that the particle shape descriptors of sphericity (*S*), roundness (*R*) and regularity (*ρ*) were assessed based on visual observation of a representative number of particles from each material through an optical microscope and the use of an empirical chart, where the regularity corresponds to the arithmetic mean of *S* and *R* (after [45]).

The polypropylene fibers consist of a polymeric material (propylene-based synthetic) with average dimensions of 12 mm in length and 0.03 mm in diameter (circular cross section), with a specific gravity of 0.9 [1,17,18]. Note that this type of fibers may find, apart from geotechnical engineering, many applications in textile science, industrial engineering and also in the improvement of structural materials [46,47,48,49,50,51,52,53,54,55].

Apart from the composite materials of the 12 different laboratory created sands with polypropylene fibers, which were used for the resonant column tests (i.e., bulk samples) to investigate the small-strain damping ratio of fiber-reinforced sand, additional microscopic (grain-scale) experiments were performed so that to provide multi-scale insights on the behavior and energy dissipation mechanisms of these composites. Specifically for the grain-scale tests, an equivalent sand (very similar to the Sydney sand but with a larger grain size) was used and it consisted of Leighton Buzzard sand (denoted as LBS). This material has origin a sedimentary rock and it consists, similar to Sydney sand, of quartz-based particles with an average size of 2 to 4 mm. Characterization of LBS particles has been presented in previous studies on sand grain contacts and sand-polymer-based contacts [56,57,58,59]. It is noted that the decision to use LBS particles for the grain-scale tests was that the micromechanical apparatus to test particle-to-particle contacts can accommodate sizes of grains, in general, between 1 and 5 mm, so it was preferable to use LBS than Sydney sand or White sand. Additionally, more spherical grains were preferable to be used for the preliminary micromechanical tests, as the coarse sized particles of Blue sand are very angular and this would make it more difficult to set the grains properly in an apex-to-apex configuration for the micromechanical tests (i.e., both LBS and Blue sand of 1–5 mm fraction would be adequate materials; however LBS are preferable particles for micromechanical tests, especially for the limited set of tests in the present study).

### 2.2. Resonant Column Tests: Sample Preparation, Experimental Setup and Testing Program

The experiments in the present study involved two different types (or scales) of tests: (i) Resonant column experiments (i.e., macroscopic tests) on bulk samples in order to assess the damping ratio of the composite fiber-sand mixtures in the range of small strains; (ii) Grain-scale experiments (i.e., microscopic tests) on pairs of sand grains with and without fibers at their contacts in order to assess their frictional behavior which can provide some additional insights (and support of the macroscopic test results) with respect to the energy dissipation mechanisms in fiber-reinforced sands. The grain-scale tests would also provide some general insights on the behavior of sand-fiber mixtures as previous works have also assessed (and modeled) the small-strain shear modulus [17,18] and the medium-strain dynamic properties [1,23] of these composite materials. It is noticed that the mainstream experimental program involved macroscopic tests, and the grain-scale tests were only supportive to the resonant column test data interpretation in the present study, as the multi-scale analysis of binary materials is a very challenging task and perhaps would require further investigation through micro-CT X-ray analysis in conjunction with discrete-based numerical analysis as mentioned earlier in the paper.

**Figure 3:**
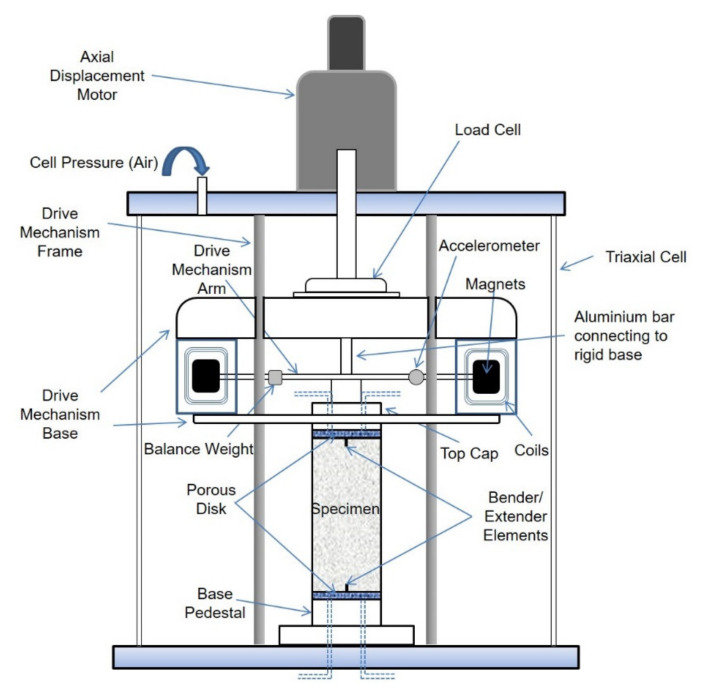
Schematic sketch of Hardin-type resonant column.

For the macroscopic tests, similar to the descriptions by Li and Senetakis [17,23] and Li et al. [1,18], the sand and the fibers were mixed at designated percentages with the addition of a small amount of water so that to distribute uniformly the fibers and accordingly, the bulk specimens were prepared in a cylindrical mold of 70 mm in diameter and 140 mm in length using the wet tamping method (note that all the specimens were compacted at a relatively high density and after the sample preparation was completed, they were fully saturated prior to the resonant column tests). A representative image of fiber-reinforced sand is given in Figure 2c. The resonant column apparatus consisted of the Hardin-type [60,61], which follows the fixed-partly fixed configuration, thus measurements of stiffness and damping are feasible on samples subjected to isotropic and anisotropic stress paths [1,22,36,60,61]. Figure 3 provides an illustration of the resonant column apparatus and its key mechanical parts. After the construction of the specimens, typical procedures of saturation and consolidation were followed and resonant column measurements were taken at different mean effective confining pressures in a range of 50 to 1000 kPa (note that the confining pressure was applied isotropically in all the experiments with typical incremental steps as 50, 100, 200, 300, 400, 500, 1000 kPa).

In total, 60 different specimens were tested in the resonant column apparatus using percentages of fibers equal to 0% (pure sand), 0.5%, 1.0%, 1.5% and 2.0%. Note that 50 (out of 60) specimens, were previously tested by Li et al. [18] and in that study details of the specimens were presented in the form of tables. Li et al. [18] presented the resonant column test data from this set of experiments in terms of small-strain stiffness, while in the present study, the data were analyzed in terms of small-strain damping ratio. Details of the additional 10 (newly constructed) specimens which were used for the complete study on 60 different samples are given in Table 2 in terms of fiber content, initial dry density and void ratio, and applied pressure. Measurements of small-strain damping ratio were performed at shear strain amplitudes in a range of 0.7 × 10^−3^ − 2.7 × 10^−3^ (%). In general, it was noticed that the addition of fibers increased the initial void ratio of the specimens and decreased their dry density.

### 2.3. Grain-Scale Tests: Sample Preparation, Experimental Setup and Testing Program

**Figure 4:**
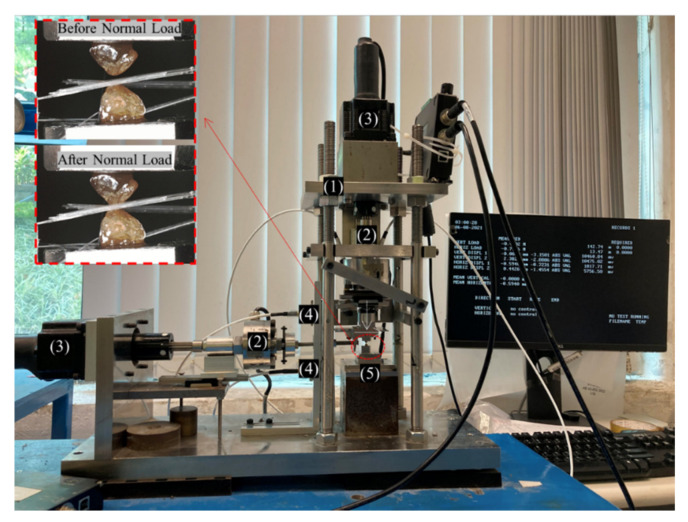
Micromechanical apparatus and the arrangement of specimen (inset). (1) Rigid frame, (2) Load cells, (3) Linear actuators, (4) non-contact displacement transducers, (5) fixed bottom grain (after [62]).

A newly designed and fabricated in-house micromechanical apparatus [62] was used to perform the grain-scale tests on LBS grains with and without the presence of fibers at their contacts. The micromechanical apparatus and its different components are shown in Figure 4. In the inset of the same figure, the arrangement of the specimens for the grain-scale shearing tests is shown. In this configuration, the two sand grains are fixed on specially designed top and bottom molds using glue and consecutively, the grains are set in contact (at a designated normal load). The displacements in the normal (vertical) and shearing (horizontal) directions are measured using robust non-contact displacement transducers with a precision of about ±0.2 μm and the corresponding mobilized loads are measured by means of bidirectional load cells with a precision of about ±0.02 N. The top grain is sheared against the fixed bottom grain (inset of Figure 4) in a displacement-controlled mode under a given constant normal load. Prior to shearing the grains at their contacts, the target normal load is applied by moving the top grain toward the fixed bottom grain.

In the present study, shearing tests were conducted under a constant normal load of 2 N up to a tangential displacement of around 60 μm. Three different pairs of grains were tested in order to assess the repeatability of the contact response for both pure sand grain contacts and contacts in the presence of polypropylene fibers (i.e., in total six pairs of grains were tested). As discussed previously, the macroscopic tests were conducted under fully saturated conditions. To replicate the similar condition in the micromechanical tests, the LBS grains were saturated with water, particularly in their contact zone using a custom-designed small-size cell. The LBS grain specimens with fibers in their contact zone were prepared by gently placing the fibers on the lower grain, and then the top grain was moved toward the bottom grain to reach the target normal load of 2N (Figure 4). The fibers were randomly oriented between the LBS grains, but they were mostly positioned parallel to the direction of shearing. The tangential (or shearing) load-displacement behavior and the corresponding mobilized interparticle friction against the tangential displacement for the cases of sand grain and sand-fiber contacts were primarily observed from the present micromechanical tests. Note that as mentioned earlier in the paper, the grain-scale tests attempted to provide only some qualitative understanding on the influence of fiber inclusion in the vicinity of the particles’ contact to infer some multi-scale insights. Thus, we did not attempt to give a direct correspondence between fiber content in the macroscopic tests and amount of fibers in the microscopic tests, but rather to infer a more general understanding on the influence of fiber inclusion at the grain-scale. These experiments can also provide basic input parameters which can be further used in discrete-based numerical simulations, for example using the discrete element method (DEM), in which case the contact response of the particles (pure sand grains or grains in the presence of fibers) constitute input parameters. Previous works on other types of geosynthetic granular materials with polymer-based inclusions (such as granulated rubber mixed with sand particles), showed that there is a direct link between grain-scale tests and bulk behavior in terms of friction as reported by Li et al. [63] and similar inferences could be made for pure granular materials as recent studies would suggest [64].

## 3. Results and Discussion

### 3.1. Small-Strain Damping Ratio of Pure Sand

**Figure 5:**
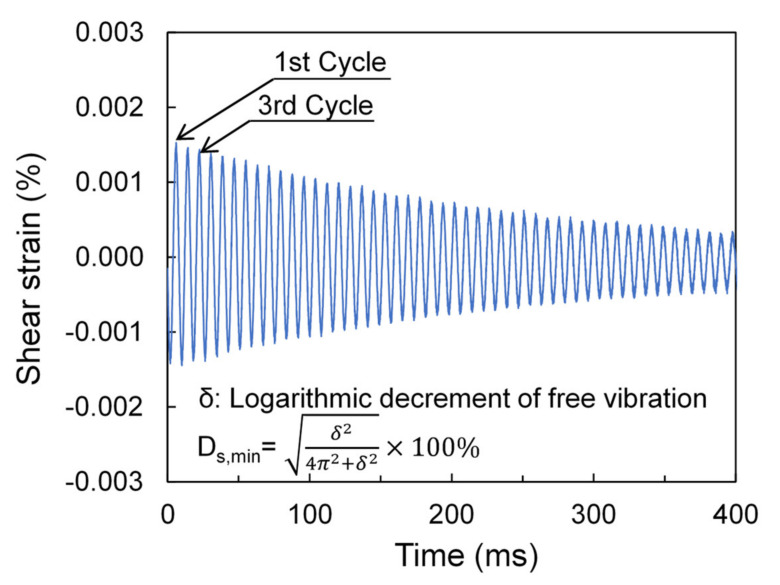
Typical test results and small-strain damping ratio calculations based on the free vibration decay method for pure SS sample at 200 kPa.

The values of small-strain damping ratio were obtained using the free vibration decay (FVD) method [65]. In the FVD method, three successive cycles during the free vibration of the samples were adopted as suggested by Stokoe et al. [27]. Note that prior to the free vibration of the specimens, they were first subjected to a frequency excitation at resonance so that to obtain the small-strain stiffness. A typical example of the experimental results along with the calculations to obtain the small-strain damping ratio using the FVD method is given in Figure 5. It is noticed that the frequency at resonance depends on different factors, such as the size of the specimen, its relative density, the magnitude of the applied confining pressure and the amplitude of the shear strain where the measurements are taken. In the present study, the resonant frequency ranged from 84 to 160 Hz for pure sand specimens, and between 62 and 141 Hz for sand-fiber mixtures and in general it was observed a decrease of the resonant frequency for higher contents of fiber and lower confining pressures. However, we would not expect any measurable influence of the resonant frequency in the obtained damping ratio values as also the study by Senetakis and Anastasiadis [66] on granular geosynthetics would suggest.

**Figure 6:**
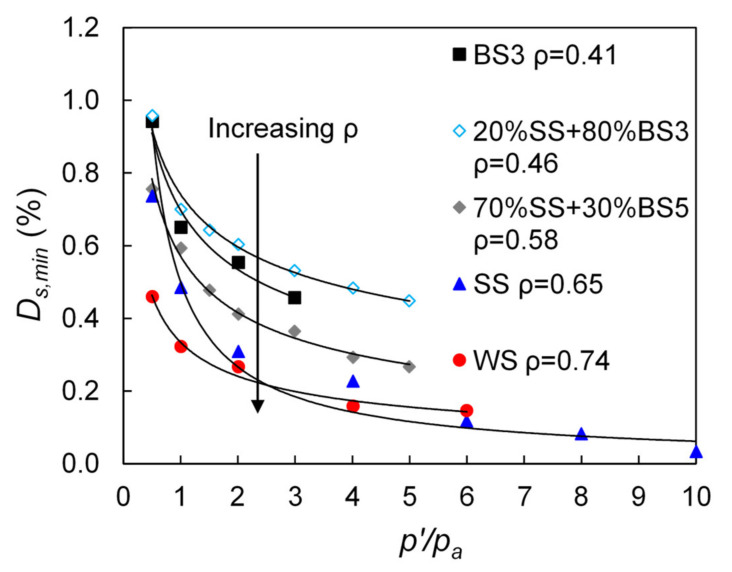
Variation of small-strain damping ratio with normalized confining pressure for five tested sands with different particle shapes.

Previous studies suggested that the small-strain damping ratio of granular materials depends, predominantly, on the confining pressure [29,31,33,34] and the particle shape [35]. Typical test results in terms of the variation of small-strain damping ratio, *Ds_,min_*, plotted against the effective confining pressure, *p*′, normalized with respect to the atmospheric pressure, *p_a_*, for pure sand specimens with different particle shapes, are presented in Figure 6. The data suggest that *Ds,_min_* decreases with increasing *p*′ and with increasing regularity (*ρ*). However, the present experiments did not show a very clear influence of the coefficient of uniformity on the *Ds,_min_* values of the pure sands. Note the significant drop of *Ds,_min_* from values in a range of, approximately, 0.45–1.00% at *p′* = 50 kPa, to values in a range of 0.20–0.50% at *p′* = 400 kPa, reaching damping ratios close to zero at the highest pressure of 1000 kPa the experiments were performed. Even though the mechanisms (and micromechanisms) contributing to dissipation of energy at very small strains are not well understood [34,67], the increase of the interparticle forces caused by the increase in the confining pressure has, perhaps, contributed to the reduction of the *Ds,_min_* values.

In general, as also the data in Figure 6 would suggest, a power-law type function can express the relationship between small-strain damping ratio with confining pressure, and its general form is given in Equation (1):

(1) $$D_{s,min}=C\times {\left(\frac{p^{\prime }}{p_{a}}\right)}^{k}$$

where *C*(%) and *k* are material parameters.

**Figure 7:**
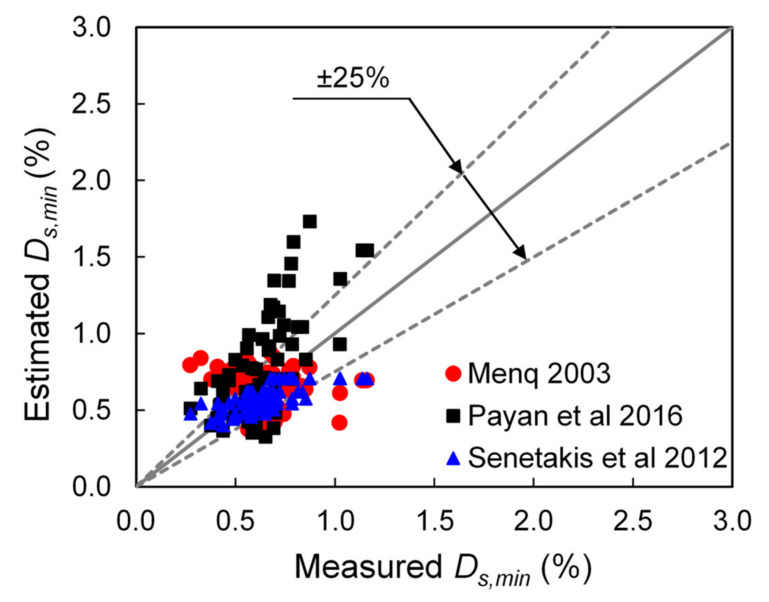
Comparison between measured small-strain damping ratio of pure sand in the current study and estimated values based on the expressions developed in the literature (Menq 2003: [29], Payan et al. 2016: [35], Senetakis et al. 2012: [31]).

Equation (1) can be considered to be a semi-analytical expression and it has been modified by Menq [29], Senetakis et al. [31], and Payan et al. [35] to incorporate the effects of grain size, gradation and particle shape characteristics, while Senetakis et al. [31] also correlated damping ratio with the geological origin of the sand, which further demonstrates that particle contact response has an influence on the macroscopic damping as observed from resonant column tests. A comparison between the measured and predicted *Ds,_min_* values of the pure sands in the current study using different expressions as presented in the literature is shown in Figure 7.

Despite the scatter in the data, the results show a satisfactory agreement between the predicted and measured small-strain damping ratios of the pure sands, with the errors to be less than 25% for the majority of the data points. In particular, the expressions proposed by Menq [29] and Senetakis et al. [31] for quartz sands provide the better prediction of the data in the present study. It is possible that the better agreement of the results in the present study with the predictive expressions by [29,31] is related with the similar size of the resonant column test samples, whereas the study by Payan et al. [35] used smaller-in-size specimens in a Stokoe-type of resonant column. As in the resonant column test, the induced voltage is controlled (which influences the applied torque to the specimen) and not the shear strain, larger-in-size specimens may be subjected to relatively lower shear strain amplitudes compared with smaller-in-size samples (because larger samples are in general stiffer) as also the study by Anastasiadis et al. [68] would suggest.

### 3.2. Small-Strain Damping Ratio of Fiber-Sand Mixtures

**Figure 8:**
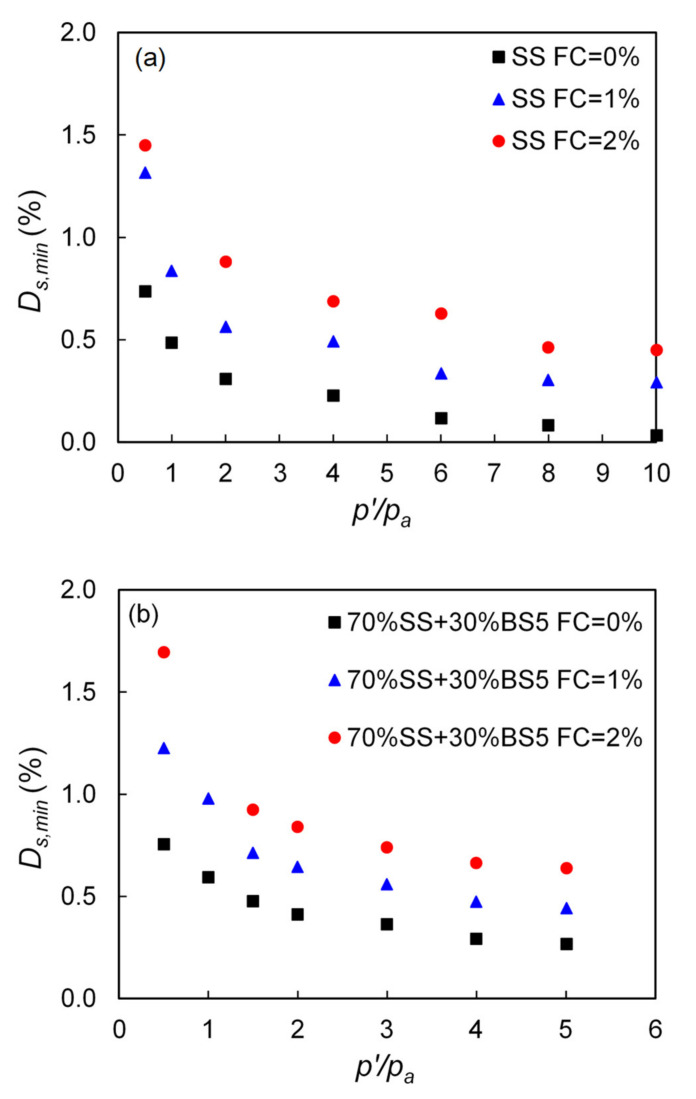
The effect of fiber content on small-strain damping ratio with two different host sands (**a**) Sydney sand (**b**) 70%SS + 30% BS5.

In the current study, and despite the scatter in the data which is commonly observed in measuring small-strain damping [29,31,34,35], the resonant column test results suggested a clear increase of small-strain damping with the increase in fiber content. Typical plots of small-strain material damping against the normalized effective confining pressure for Sydney sand and Sydney sand—Blue sand mixtures (70% SS and 30% BS5) with varying fiber content are shown in Figure 8a,b. It is noticed in Figure 8a that for the pure Sydney sand (0% of fiber content), *Ds,_min_* was equal to about 0.3% at *p’* = 200 kPa, but for contents of fiber between 1–2%, material damping had values between about 0.6% and 0.9% and a similar trend was also observed for the 70%SS + 30% BS5 sand in Figure 8b. These results are representative of the total set of experiments for which material damping was measured.

**Figure 9:**
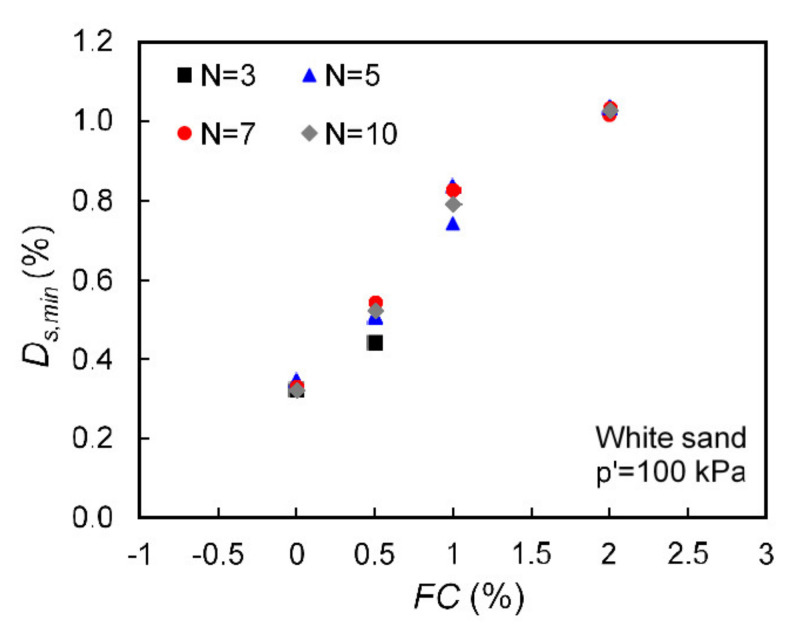

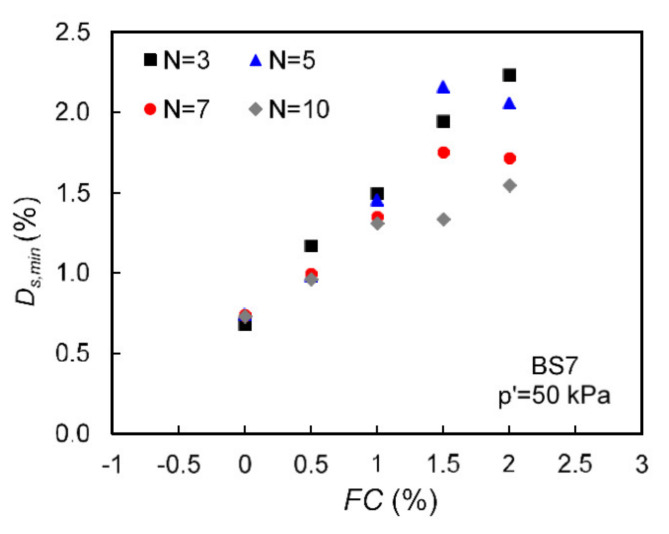
Small-strain damping ratio obtained from different number of cycles (N) used to compute the logarithmic decrement from the free decay curve for specimens with varying fiber content.

Plots illustrating the influence of the content of fiber and the number of cycles used to interpret damping for representative groups of samples are given in Figure 9. These results indicate an increase of material damping at small strains with an increase of fiber content no matter the number of cycles used to calculate damping ratio. Note that the intention of presenting the results using different number of cycles in Figure 9 was solely to provide roughly a range of values for material damping accounting for the effect of the total number of cycles used during the free vibration exercise. Even though for the majority of the specimens there was not observed any significant influence of the number of cycles used in the FVD analysis to interpret damping, some influence was observed only at higher fiber contents (1.5–2.0%) and when the D_s,min_ values were higher (implying that the data were obtained at lower confining pressures), which is perhaps related with the much lower resonant frequencies corresponding to these data.

### 3.3. Development of a New Expression for Small-Strain Damping Ratio of Fiber-Sand Mixtures

**Figure 10:**
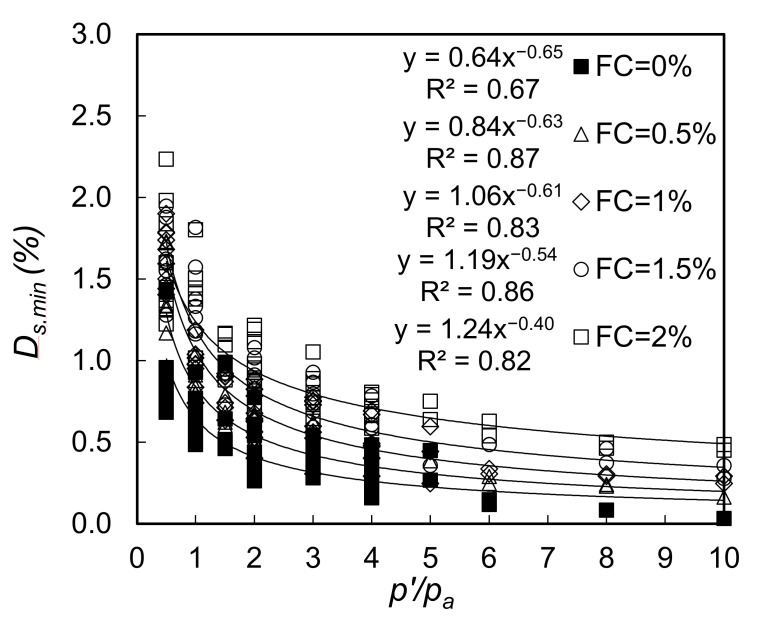
Variation of small-strain damping ratio with normalized confining pressure for all tested sands with different fiber content.

**Figure 11:**
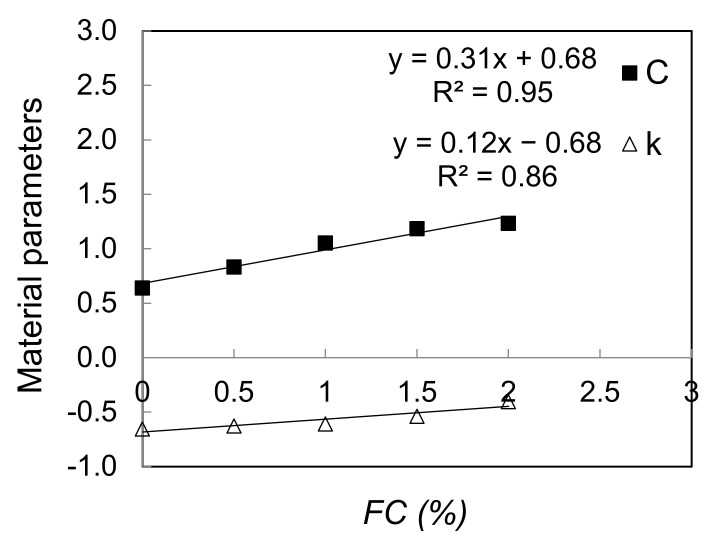
The effect of fiber content on material damping parameters *C* and *k*.

To quantify the effect of fiber content on damping ratio values, a power-law type expression (Equation (1)) has been fitted to all the experimental data, as shown in Figure 10. Using the least square error approach, the best fitting constants C and k for each given fiber content have been obtained as also depicted on the same figure. For example, for fiber content of 0%, C equals to 0.64% and k equals to 0.65. ‘*C*’ and ‘*k*’ values obtained from the whole set of experimental data are plotted against fiber content (*FC*) in Figure 11.

The best linear trend using the minimum least square error with a coefficient of determination of R^2^ = 0.95 and R^2^ = 0.86 for ‘*C*’ and ‘*k*’ respectively, were then obtained to describe *C* and *k* as a function of *FC* as follows:

(2) C=0.31×FC+0.68

(3) k=0.12×FC−0.68

where *FC* is expressed as (%).

Based on the analysis described above and the estimation of the model parameters *C* and *k*, the following expression is proposed for the prediction of the *Ds,_min_* values of fiber-sand mixtures:

(4) $$D_{s,\min}\left(\%\right)=\left(0.31\times FC+0.68\right)\times {\left(\frac{p^{\prime }}{p_{a}}\right)}^{0.12\times FC-0.68}$$

**Figure 12:**
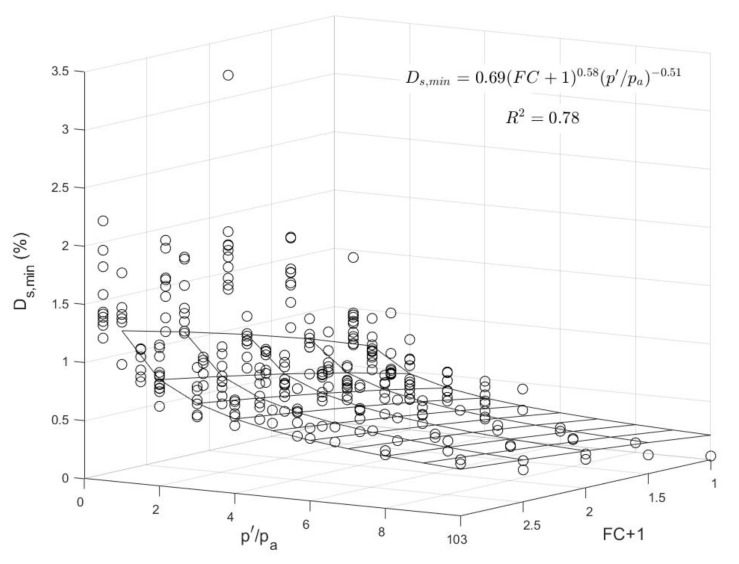
Curve-fitting of small-strain damping ratio against fiber content and normalized effective pressure.

A second attempt was made to correlate fiber content and normalized effective stress with small-strain damping ratio directly using a three-dimensional surface-fitting. Figure 12 gives this correlation in terms of a three-dimensional plot. Based on regression analysis using the least square method, the following expression was derived for *Ds,_min_*:

(5) $$D_{s,min}\;\left(\%\right)=0.69\times {\left(FC+1\right)}^{0.58}\times {\left(\frac{p^{\prime }}{p_{a}}\right)}^{-0.51}$$

**Figure 13:**
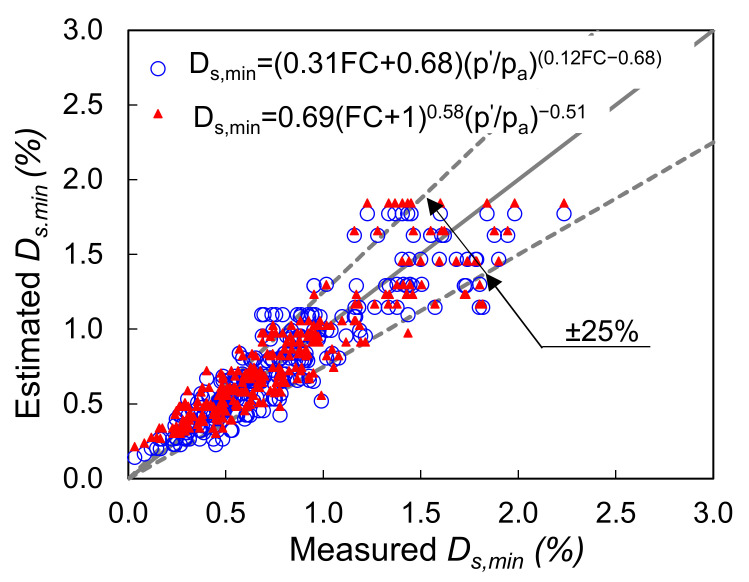
Comparison between measured and estimated small-strain damping ratio.

Based on the developed model of Equations (4) and (5), Figure 13 gives a comparison between predicted and measured values of small-strain damping ratio. These results demonstrated a good prediction of the data within a scatter of ±25% and that the estimated values of *Ds,_min_* obtained using Equations (4) and (5) are relatively close in magnitude. Expressions in Equation (4) or Equation (5), can be used for the complete modeling of the damping ratio of sand-fiber mixtures in conjunction with the normalized medium-strain developed expressions for damping ratio as proposed by [1].

### 3.4. Multi-Scale Insights

**Figure 14:**
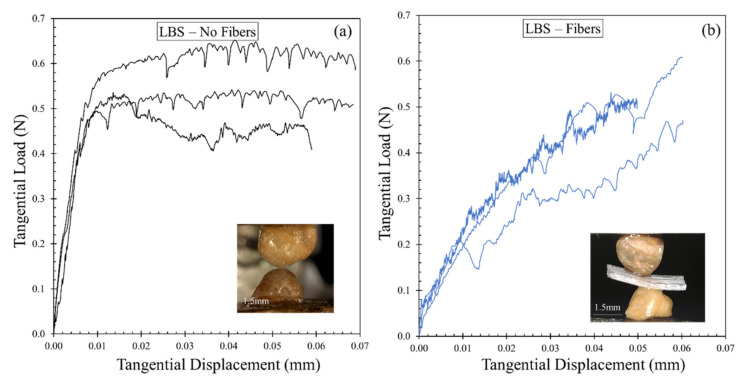
Grain-scale test results: Tangential load—displacement of (**a**) Pure sand particle contacts. (**b**) Sand particle contacts in the presence of fibers.

**Figure 15:**
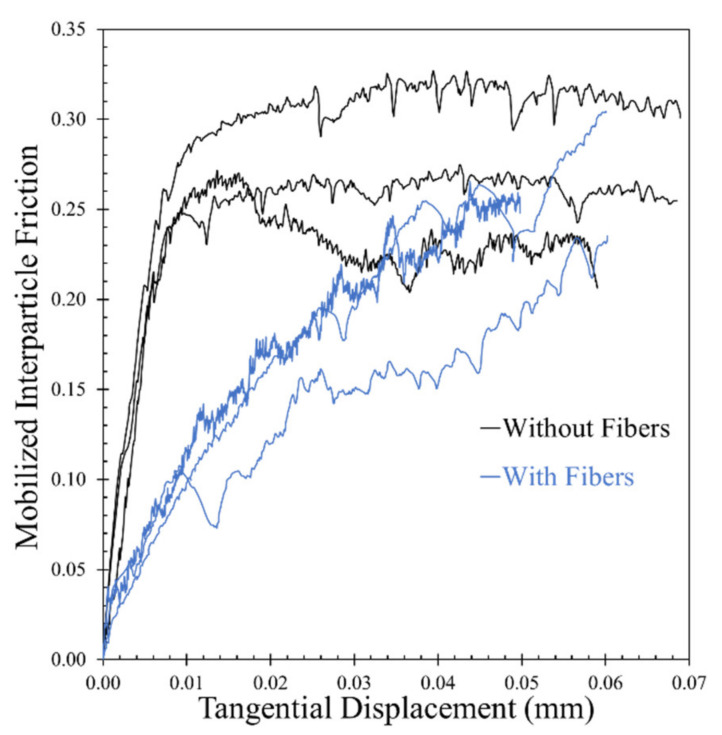
Grain-scale test results in terms of mobilized coefficient of (interparticle) friction against displacement.

It was not intended in the present study to provide a quantitative correlation between the macroscopic experiments (small-strain damping ratio of bulk specimens) with the observed behavior from the grain-scale tests (frictional response of the grains at their contacts) as also discussed earlier, but rather to give some qualitative understanding of the influence of fiber inclusion at micro- and macroscopic levels, without extrapolating the micromechanical test results in a direct quantitative analysis of the bulk samples. Figure 14 gives a summary of the results from the micromechanical tests in terms of tangential load against displacement for pure sand grain contacts (Figure 14a) and sand grain contacts in the presence of fibers (Figure 14b), while the same data in terms of mobilized interparticle friction against displacement are given in Figure 15.

For the pure sand grain contacts, it is observed that the curves reach a steady-state sliding (despite some fluctuations of the load influenced by the morphology of the grains) at displacements in the range of, approximately, 10 to 20 μm. Initially, the tangential load increased nonlinearly (indicating a gradual reduction of tangential stiffness) reaching a steady-state sliding, where tangential stiffness drops to zero; this threshold is termed as the slip displacement and it depends on material type (hardness, Young’s modulus, morphology) as well as the normal load magnitude [57].

For the sand grain contacts in the presence of fibers, a very different response was observed, with a continuous increase of the tangential load (and the mobilized friction) with increasing displacement without any observation of a steady-state regime to be reached for the composite specimens which also implies extended slip displacements to be necessary in order to mobilize a steady-state sliding that was beyond the applied displacements in these experiments. As the curves in Figure 15 are normalized plots of tangential load over the normal load and the normal load was equal in all the experiments, the slopes of the curves in their initial regime also provide an indication of the tangential stiffness. These data suggested a reduction of the tangential stiffness when fibers inferred at the contact of the sand particles.

Previous studies based on macroscopic tests on fiber-reinforced sand showed that in general, small-strain stiffness decreases when fibers are added in the solid matrix [17,18]; this behavior may have been contributed by the reduced contact stiffness of the sand particles because of the presence of fibers. In addition, the studies by Li and Senetakis [23] and Li et [1] showed that the inclusion of fibers results in slower stiffness reduction at the macroscopic level, which also depicts a slower damping increase rate for fiber-sand mixtures compared with pure sand in the regime of medium strains; these observations have also been reported for other types of elastomeric materials such as granulated rubber mixed with sand [69]. These observations may have been contributed to by the higher linearity of the tangential load—displacement curves of the sand-fiber-sand contacts as observed from the grain-scale experiments in the present study. For the small-strain damping ratio, it is more difficult to provide a direct link between micro- and macroscopic experimental results as it is not straightforward to interpret the grain-scale displacements which correspond precisely to the perturbations from the resonant column torsional excitation; however, the sand grain contacts (without fibers) have in general higher friction compared with that of fibers (at least for the range of displacements the micromechanical tests were performed). This infers that damping ratio at small-strains and small-to-medium strains may have different contributing mechanisms. At small-to-medium strains, where the macroscopic behavior is highly nonlinear, the damping ratio (or damping ratio increase rate) is, predominantly, hysteretic and is directly linked with the (macroscopic) stiffness reduction curves. Higher linearity at the grain-scale, or alternative, extended slip displacements to reach a steady-state sliding at the contacts of the particles, generally leads to slower stiffness reduction and subsequently slower damping increase rate. At small-strains, for the sand particle contacts, the energy is assumed to be dissipated through damage of micro-asperities (and perhaps some very small amount of heat caused by the frictional resistance at the grain-scale). This, in turn, may result in smaller energy dissipation of the bulk samples, which in general agrees with the qualitative interpretations made in the study by Senetakis et al. [32]. On the other hand, less microscopic energy dissipation (as hysteretic mechanism), may lead to the opposite result at the macroscopic level leading to increased bulk damping in the resonant column tests of fiber-sand mixtures. Especially for composite interfaces of sand particles sliding on polymeric surfaces, similar to that of sand-rubber composite systems, the response of sand-fiber-sand interfaces may have also been contributed by viscous mechanisms which cause an increased bulk energy dissipation [59,70]. In general, at small-strain perturbations, the results in the present study suggest a negative correlation between bulk damping and interparticle friction (which in turn, is related with the extended slip displacements in fiber-sand composite interfaces).

## 4. Conclusions

A set of resonant column tests has been performed on sand with different particle shapes and gradations mixed with various contents of fibers, to determine the small-strain damping ratio of fiber-sand mixtures. The test results indicated that the presence of fibers led to an increase of small-strain damping ratio. Using a power-law type of fitting, the effect of fiber content was isolated and incorporated into the development of a new expression for the determination of small-strain damping ratio for fiber-sand mixtures subjected to isotropic confining stress. Comparisons of predicted values of small-strain damping ratio based on the new model and the data reported in this study demonstrate a satisfactory performance of the new expression. A three-dimensional curve-fitting equation was also developed to make additional comparisons. In general, these two equations were shown to give relatively closely predicted *Ds,_min_* values. In general, based on the macroscopic test results, the increase of damping ratio with fiber content may provide a promising direction in applications in which the target is to mitigate induced seismic and vibration loads as the fibers provide a means of energy dissipation to the composite material. Multi-scale analysis on the influence of fiber inclusion on the bulk behavior of fiber-sand composite mixtures was further elaborated performing a limited number of grain-scale tests on particle-to-particle contacts (sand grains) with and without fibers. It was attempted to interpret the macroscopic behavior of the samples, both in terms of small-strain damping ratio (results from the present study) and also in terms of small-strain stiffness and medium-strain behavior as previous studies reported on fiber-sand composite materials. The results demonstrated a negative correlation between bulk small-strain damping ratio with interparticle friction and that extended displacement are necessary in the grain-scale tests to obtain a steady-state sliding when fibers are added. This could explain the slower stiffness reduction and damping increase curves of fiber-sand mixtures as obtained in resonant column tests. The addition of fibers at the contacts of the sand grains also reduced the contact stiffness which might provide some explanations on the reduced macroscopic stiffness of fiber-sand mixtures compared with that of pure sand.

## Figures and Tables

**Table 1:** Basic Properties of tested soils.

Sand Type	Sand Name	Grain Size Distribution			Particle Shape Descriptors *		
		*d_50_*(mm)	*Cu*	*Cc*	*R*	*S*	*ρ*
Sydney Sand	SS	0.33	2.18	0.89	0.63	0.68	0.65
Blue Sand 1	BS 1	0.99	5.84	1.22	0.28	0.54	0.41
Blue Sand 2	BS 2	0.96	2.98	0.88	0.28	0.54	0.41
Blue Sand 3	BS 3	1.00	1.66	0.90	0.28	0.54	0.41
Blue Sand 4	BS 4	3.35	1.42	0.93	0.28	0.54	0.41
Blue Sand 5	BS 5	0.42	1.41	0.93	0.28	0.54	0.41
Blue Sand 6	BS 6	1.67	1.41	0.93	0.28	0.54	0.41
Blue Sand 7	BS 7	2.5	2.08	0.89	0.28	0.54	0.41
50% SS + 50% BS 3	50SS-50BS3	0.63	3.75	0.91	0.46	0.61	0.53
20% SS + 80% BS 5	20SS-80BS5	0.41	1.47	0.93	0.35	0.57	0.46
White sand	WS	0.24	1.66	0.90	0.71	0.76	0.74
70% SS + 30% BS5	70SS-30BS5	0.37	2.2	1.18	0.53	0.64	0.58

* *R*: Roundness, *S*: Sphericity, *ρ*: Regularity.

**Table 2:** Testing program and specimen details (additional tests are presented here, the majority of the samples used in the study were presented in the form of tables by [18]).

Sample No.	Sand Type	Fiber Content (%)	Initial Dry Density γ_d_ (kN/m^3^)	Initial Void Ratio (e)
1	WS	0	16.74	0.553
2	WS	0.5	15.03	0.733
3	WS	1	14.72	0.732
4	WS	1.5	14.14	0.787
5	WS	2	13.94	0.796
6	70SS-30BS5	0	16.18	0.607
7	70SS-30BS5	0.5	14.50	0.776
8	70SS-30BS5	1	13.92	0.833
9	70SS-30BS5	1.5	13.49	0.874
10	70SS-30BS5	2	13.38	0.872

## Data Availability

Data are available by the corresponding author after reasonable request.
